# Disease-Causing 7.4 kb *Cis*-Regulatory Deletion Disrupting Conserved Non-Coding Sequences and Their Interaction with the *FOXL2* Promotor: Implications for Mutation Screening

**DOI:** 10.1371/journal.pgen.1000522

**Published:** 2009-06-19

**Authors:** Barbara D'haene, Catia Attanasio, Diane Beysen, Josée Dostie, Edmond Lemire, Philippe Bouchard, Michael Field, Kristie Jones, Birgit Lorenz, Björn Menten, Karen Buysse, Filip Pattyn, Marc Friedli, Catherine Ucla, Colette Rossier, Carine Wyss, Frank Speleman, Anne De Paepe, Job Dekker, Stylianos E. Antonarakis, Elfride De Baere

**Affiliations:** 1 Center for Medical Genetics, Ghent University Hospital, Ghent, Belgium; 2 Department of Genetic Medicine and Development, University of Geneva Medical School, Geneva, Switzerland; 3 Program in Gene Function and Expression and Department of Biochemistry and Molecular Pharmacology, University of Massachusetts Medical School, Worcester, Massachusetts, United States of America; 4 Division of Medical Genetics, Royal University Hospital, Saskatoon, Saskatchewan, Canada; 5 Reproductive Endocrine Unit, Hôpital Saint-Antoine, Paris, France; 6 Royal North Shore Hospital, Sydney, Australia; 7 Department of Clinical Genetics, The Children's Hospital at Westmead, Westmead, Australia; 8 Department of Ophthalmology, Justus-Liebig-University Giessen, Universitaetsklinikum Giessen und Marburg GmbH Giessen Campus, Giessen, Germany; University of Washington, United States of America

## Abstract

To date, the contribution of disrupted potentially *cis*-regulatory conserved non-coding sequences (CNCs) to human disease is most likely underestimated, as no systematic screens for putative deleterious variations in CNCs have been conducted. As a model for monogenic disease we studied the involvement of genetic changes of CNCs in the *cis*-regulatory domain of *FOXL2* in blepharophimosis syndrome (BPES). Fifty-seven molecularly unsolved BPES patients underwent high-resolution copy number screening and targeted sequencing of CNCs. Apart from three larger distant deletions, a *de novo* deletion as small as 7.4 kb was found at 283 kb 5′ to *FOXL2*. The deletion appeared to be triggered by an H-DNA-induced double-stranded break (DSB). In addition, it disrupts a novel long non-coding RNA (ncRNA) *PISRT1* and 8 CNCs. The regulatory potential of the deleted CNCs was substantiated by *in vitro* luciferase assays. Interestingly, Chromosome Conformation Capture (3C) of a 625 kb region surrounding *FOXL2* in expressing cellular systems revealed physical interactions of three upstream fragments and the *FOXL2* core promoter. Importantly, one of these contains the 7.4 kb deleted fragment. Overall, this study revealed the smallest distant deletion causing monogenic disease and impacts upon the concept of mutation screening in human disease and developmental disorders in particular.

## Author Summary

Long-range genetic control is an inherent feature of genes harbouring a highly complex spatiotemporal expression pattern, requiring a combined action of multiple *cis*-regulatory elements such as promoters, enhancers, and silencers. Consequently, disruption of the long-range genetic control of a target gene by genomic rearrangements of regulatory elements may lead to aberrant gene transcription and disease. To date, the contribution of mutated regulatory elements to human disease has not been studied frequently. Here, we explored the contribution of genetic changes in potentially *cis*-regulatory elements of the *FOXL2* gene in blepharophimosis syndrome (BPES), a developmental monogenic condition of the eyelids and ovaries. We identified a *de novo* very subtle deletion of 7.4 kb causing BPES. Moreover, we studied the functional capacities and chromosome conformation of the deleted region in *FOXL2* expressing cellular systems. Interestingly, the chromosome conformation analysis demonstrated the close proximity of the 7.4 kb deleted fragment and two other conserved regions with the *FOXL2* core promoter, and the necessity of their integrity for correct *FOXL2* expression. Finally, our study revealed the smallest distant deletion causing monogenic disease and emphasized the importance of mutation screening of *cis*-regulatory elements in human genetic disease.

## Introduction

Many recent studies have provided insights into the biological relevance of the non protein-coding portion of the human genome, previously referred to as junk DNA. One of them is the ENCODE pilot study, which has revealed that the number of functional genomic elements is much higher than previously anticipated, and that the vast majority of elements regulating gene expression are contained in the non-protein coding portion of the genome. In addition, it shed light on the pervasively transcribed nature of the human genome [1].

Comparative analysis of genomes is a major tool for the identification of regulatory elements. In this context, several arbitrary criteria have been used to define evolutionarily conserved elements, such as conserved non-coding sequences (CNCs) that were originally defined as elements sharing ≥70% homology over ≥100 bp of ungapped alignment of human and mouse sequences [2]–[4]. A fraction of them (i.e. the most conserved ones) have been shown to function as *cis*-regulatory elements, predominantly controlling the spatiotemporal expression of developmental genes [5]–[7]. To date, the contribution of disrupted potentially regulatory CNCs to human genetic disease is most likely underestimated, as no systematic screens for putative deleterious variations in CNCs have been conducted in this respect. One of the reasons for this is the large extent of the regions to be investigated, as the regulatory domain of a gene can stretch beyond 1 Mb in both directions of its transcription unit. In addition, putative functional consequences of variations outside a transcription unit are difficult to assess.

An example of a developmental gene with a strictly regulated spatiotemporal expression pattern is *FOXL2* (NM_023067). It is known to be the disease-causing gene of blepharophimosis-ptosis-epicanthus inversus syndrome (BPES) [MIM 110100], a rare autosomal dominant development disorder of the eyelids with (BPES type I) or without (BPES type II) premature ovarian failure (POF) [8]. Overall, sporadic and familial BPES can be explained by intragenic mutations and gene deletions in 71% and 11% of the patients respectively [9]. Interestingly, we identified microdeletions upstream and downstream of *FOXL2* in 4% of BPES [9],[10]. In addition, 3 translocation breakpoints upstream of *FOXL2* have been described [8],[11],[12]. Until now, there is no evidence for genetic heterogeneity of this condition. From the 5 reported microdeletions outside *FOXL2*, one is located 3′ to *FOXL2*, while the others are located 5′ to *FOXL2* and share a smallest region of deletion overlap (SRO) of 126 kb [10]. This SRO is located 230 kb upstream of *FOXL2*, telomeric to the three previously characterized translocation breakpoints, and contains several CNCs, harbouring putative transcription factor binding sites. Moreover, the SRO contains the human orthologue of the Polled Intersex Syndrome (PIS) mutation in goat. The PIS goat is a natural animal model for BPES associating absence of horns (polledness) and intersexuality. The sex reversal exclusively affects female animals in a recessive manner, whereas the absence of horns is dominant in both sexes. The phenotype is caused by a regulatory 11.7 kb deletion located 280 kb upstream of goat *FOXL2*. It was shown that the deletion alters the transcription of at least three genes: *FOXL2*, the non-protein coding gene *PISRT1* (PIS-regulated transcript 1) (AF404302) and *PFOXic* (promoter *FoxL2* inverse complementary) (AY648048) [13]–[15]. In agreement with the findings in the translocation patients and in the PIS goat, the distant microdeletions found in human BPES were hypothesized to disturb long-range transcriptional control of *FOXL2* expression through the disruption of one or more *cis*-acting regulatory elements. These findings added to an increasing number of long-range genetic defects in human development conditions [16]–[19].

Apart from translocations and microdeletions/duplications of *cis*-regulatory elements, subtle copy number variations (CNVs) or sequence variations of *cis*-regulators can also be associated with a phenotype in humans. These have been found in the long-range limb-specific *cis*-regulatory element ZRS of the *SHH* gene (NM_000193), leading to preaxial polydactyly (PPD) (PPD2, MIM 174500), isolated triphalangeal thumb (MIM 174500), and triphalangeal thumb-polysyndactyly (TPTPS) phenotypes (MIM 174500) [20]–[23]. In addition, Benko et al. reported a heterozygous point mutation in a highly conserved non-coding conserved sequence located 1.44 Mb upstream of *SOX9* in a patient with Pierre Robin sequence (PRS, OMIM 261800) [19].

To date, the underlying molecular defect remains unknown in 12% of BPES patients [9]. Here, we focus on the contribution of previously unidentifiable and subtle deletions/duplications, and sequence variations in putative *cis*-regulatory elements surrounding *FOXL2* in BPES. We developed a combined strategy consisting of microarray based comparative genome hybridization (array CGH), high-resolution quantitative PCR (qPCR) and sequencing of CNCs located in the SRO 5′ to *FOXL2*. Samples from 57 BPES patients who do not carry an intragenic *FOXL2* mutation or gene deletion were studied, revealing a distant 7.4 kb deletion as the most prominent finding. The deletion harbours putative regulatory elements. Functional studies in cellular systems were performed to assess their regulatory potential. In addition, Chromosome Conformation Capture analysis (3C) was conducted to provide insights into the spatial organisation and interaction patterns of a normal and a disrupted *FOXL2* locus.

## Results/Discussion

**Figure 1:**
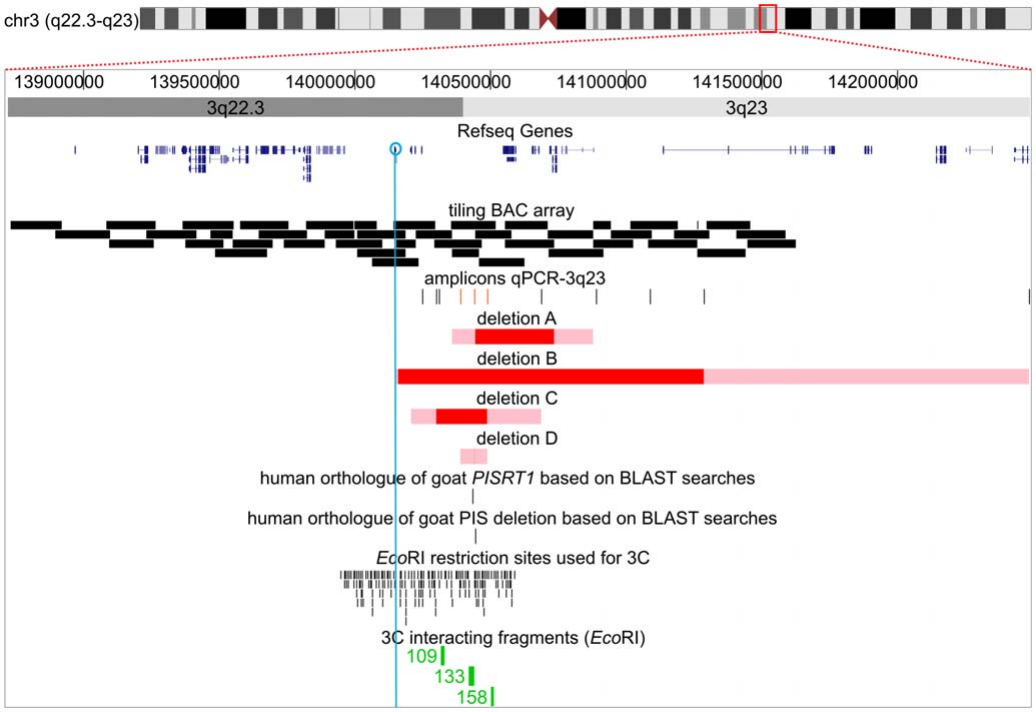
Human Genome Browser view of the *FOXL2* region. The *FOXL2* region (chr3:138,720,000–143,780,300) with custom tracks showing the BACs and qPCR-3q23 amplicons used in the study. Locations and sizes of the novel deletions are indicated by horizontal bars. The red bars indicate the minimal deleted regions and the pink bars indicate the regions harbouring deletion breakpoints. Two tracks indicate the human orthologous regions of caprine *PISRT1* and the caprine PIS deletion based on BLAST searches. The last two tracks at the bottom represent the *Eco*RI restriction sites used for 3C and the 3C fragments interacting with the fragment containing the *FOXL2* promotor (in green). The figure was drawn according to the UCSC, Human Genome Browser, March 2006.

**Figure 2:**
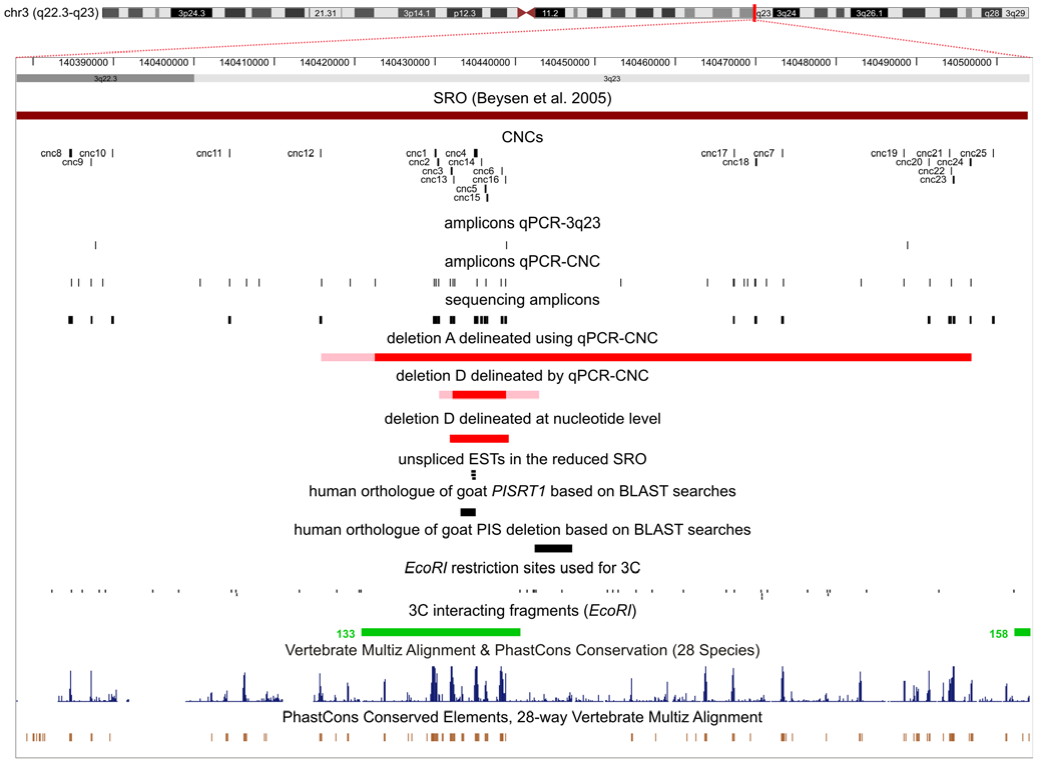
Human Genome Browser view of the initial and reduced SRO. UCSC Genome Browser view of the SRO region (chr3:140,377,900–140,504,100) with: 25 CNCs mapping within the initial SRO; 3 amplicons of the qPCR-3q23 assay located in the initial SRO; 36 amplicons designed for the qPCR-CNC assay; the amplicons designed for sequencing analysis of CNCs and the two deletions delineated by qPCR-CNC (A,D). The red bars indicate the minimal deleted regions and the pink bars indicate the maximum deleted region. The next track indicates the delineation of deletion D at nucleotide level. The remaining tracks at the bottom display: unspliced ESTs in the reduced SRO; the human orthologue of caprine *PISRT1* based on BLAST searches; the human orthologue of the PIS deletion based on BLAST searches; the *Eco*RI restriction sites used for 3C; in green the 3C fragments (133, 158) interacting with the fragment containing the *FOXL2* promotor; the conservation profile of the region extracted from the UCSC genome browser (blue and brown). The figure was drawn according to the UCSC, Human Genome Browser, March 2006.

Comparative analysis of genomes is a major tool for the identification of regulatory elements [2]. In this context, a comparative analysis of the human and mouse orthologous regions spanning the SRO revealed 25 CNCs with an average length of 165 bp and average homology of 82.5% (Table 1). These identified CNCs were an important focus here. We included 57 patients with a diagnosis of BPES who tested negative for intragenic mutations and copy number changes of *FOXL2*. First, these patients were screened for copy number changes outside *FOXL2*, with special interest for the initial SRO region of upstream deletions. This was carried out by one or a combination of the following assays: microsatellite analysis, arrayCGH and two qPCR assays called qPCR-3q23 (Figure 1) and qPCR-CNC (Figure 2) respectively. The use of different techniques can be explained by the availability of more convenient techniques in the course of the study. In a second step, the remaining negative patients were specifically screened for sequence variants of CNCs within the initial SRO (Figure 2). In addition, functional analyses (i.e. luciferase assays) were performed for wild type and variant CNCs in different cellular systems. Finally, the chromosome conformation of the *FOXL2* locus was investigated by 3C.

**Table 1:** Mapped CNCs within the initial SRO.

CNC	#Chr	Start (hg18)	Stop (hg18)	Length (bp)	% id. Hs-Mm	% id. Hs-Galgal	Beysen et al. 2005 [10]	Crisponi et al. 2004 [69]	Reduced SRO (Figure 2 and 4)	3C (Figures 1, 2 and 5; Figure S2)
CNC8	chr3	140384474	140384783	310	82,3	no match				
CNC9	chr3	140387170	140387278	109	87,2	no match				
CNC10	chr3	140389814	140389935	122	76,2	no match				
CNC11	chr3	140404369	140404478	110	71,8	no match				
CNC12	chr3	140415708	140415847	140	80,0	no match				
CNC1	chr3	140430011	140430163	153	85,0	33,3	CNG3	9 kb conserved block		interacting fragment 133
CNC2	chr3	140430359	140430589	231	75,8	50,2	CNG2	9 kb conserved block		interacting fragment 133
CNC3	chr3	140431968	140432183	216	99,1	94,0	CNG4	9 kb conserved block	7.4 kb deletion	interacting fragment 133
CNC13	chr3	140432327	140432425	99	80,8	few bases		9 kb conserved block	7.4 kb deletion	interacting fragment 133
CNC4	chr3	140434920	140435316	397	94,2	71,5	CNG5	9 kb conserved block	7.4 kb deletion	interacting fragment 133
CNC14	chr3	140435755	140435885	131	80,2	no match		9 kb conserved block	7.4 kb deletion	interacting fragment 133
CNC5	chr3	140436196	140436391	196	87,2	61,7	CNG6	9 kb conserved block	7.4 kb deletion	interacting fragment 133
CNC15	chr3	140436467	140436614	148	72,3	no match		9 kb conserved block	7.4 kb deletion	interacting fragment 133
CNC6	Chr3	140438276	140438409	134	78,4	75,4	CNG7	9 kb conserved block	7.4 kb deletion	interacting fragment 133
CNC16	chr3	140438782	140438885	104	80,8	no match		9 kb conserved block	7.4 kb deletion	interacting fragment 133
CNC17	chr3	140467213	140467325	113	85,0	no match				
CNC18	chr3	140469910	140470106	197	72,6	no match				
CNC7	chr3	140473283	140473397	115	92,2	73,9	CNG9			
CNC19	chr3	140488450	140488549	100	86,0	no match				
CNC20	chr3	140491469	140491687	219	84,0	no match				
CNC21	chr3	140494100	140494210	111	86,5	no match				
CNC22	chr3	140494313	140494442	130	86,2	no match				
CNC23	chr3	140494476	140494759	284	91,9	no match				
CNC24	chr3	140496676	140496848	173	78,0	no match				
CNC25	chr3	140499555	140499663	109	73,4	no match				

All CNCs overlap with PhastCons (28 species, UCSC table, hg18).

Abbreviations: chr.: chromosome; id.: identity; Hs: *Homo sapiens*; Mm: *Mus musculus*; Galgal: *Gallus gallus*.

ArrayCGH revealed 1 novel extragenic deletion 5′ to *FOXL2* which was further delineated by qPCR-CNC at the centromeric end (Deletion A) (Figure 1 and Figure 2). In addition, qPCR-3q23 with 3 amplicons located in the SRO revealed 3 more novel extragenic deletions (Deletion B–D). Deletion B and C, both encompassing all 3 amplicons and identified in typical BPES patients, were subsequently further delineated using additional amplicons (Figure 1). Deletions B and C were found to be 190 kb–478 kb and 1.12 Mb–2.3 Mb in size respectively (Figure 1). Deletions A, B and C can be added to the previously described relatively large deletions 5′ to *FOXL2*, which were believed to be pathogenic through the deletion of *cis*-regulatory elements [10]. Here, the *de novo* occurrence could be assessed for deletion C for which parental DNA was available.

Most remarkable, however, was the identification of the very subtle deletion D, which encompassed only 1 amplicon. Deletion D could be mapped to a region of minimum 6 kb and maximum 12.5 kb in size using qPCR-CNC. Subsequent long-range PCR and direct sequencing of the junction PCR fragment allowed us to define its extact size (7358 bp) and location (chr3:140,431,841-140,439,199), being 283 kb upstream of *FOXL2* (Figure 2). This deletion is entirely retained within the previously described SRO of 126 kb and thus defines a drastically reduced SRO. Furthermore, segregation analysis suggested a *de novo* occurrence of this small deletion, sustaining its pathogenic potential. Despite its small size, the deletion is presumed to lead to a classic BPES phenotype in a 7-year-old sporadic male.

**Figure 3:**
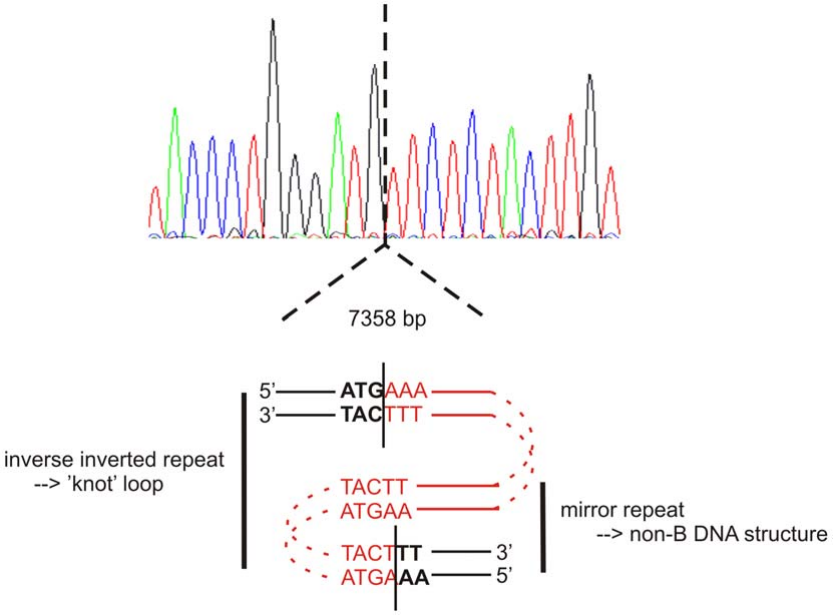
Characterization of 7.4 kb deletion. Top: Sequence electropherogram of the junction fragment of deletion D. The dotted vertical line indicates the position of the junction. Bottom: Schematic representation of the proposed mechanism underlying the 7.4 kb deletion. The deleted fragment is delineated by the vertical lines and represented in red. The retained basepairs are formatted in bold. It is hypothesized that a double-stranded break (DSB) at the telomeric side triggered the deletion, followed by a DSB repair mechanism guided by the formation of a knot loop between the reverse complement of the pentanucleotide motif at the centromeric end.

The observation that all known and novel regulatory deletions do not show recurrent breakpoint regions argues against non-allelic homologous recombination (NAHR) as a possible mechanism underlying this subtle deletion [24]. Other models such as non-homologous end joining (NHEJ) or Forkhead Stalling and end Switching (FoSTeS) might explain the formation of the deletion, although there is no scar at the junction fragment [24]. To unravel the mechanism responsible for this deletion, bioinformatics analyses of the breakpoint junctions was performed. A 70 bp ClustalW alignment of the abnormal junction sequence with the reference genomic sequence from both breakpoint regions, did not reveal any significant homologies, although there is some minor sequence similarity. Similarly, BLAST2 analysis of the 2 kb breakpoint regions did not reveal any significant similarities either. Analysis with RepeatMasker indicated a 36-bp low complexity region at the centromeric end of the deletion, but no additional repetitive elements. At the telomeric end it revealed a LINE2 repeat in very close proximity of the breakpoint and at a larger distance a 25-bp simple repeat and a 123-bp low complexity region. Tandem repeats and palindromes were excluded in a region of 300 bp around the breakpoints using Mreps and Palindrome. In addition, the GC content of a 1-Mb region around the 7.4 deletion appeared not to be above average. Interestingly, with DNA Pattern Finder three motifs, known to be implicated in DNA rearrangements elsewhere, were identified in 70-bp regions surrounding the breakpoints, including one of the immunoglobulin heavy chain class switch repeats (GGGCT), a deletion hotspot consensus site (TG[AG][AG][GT][AC]) and a DNA polymerase α pause site core sequence GC [GC]. It cannot be excluded, however, that the occurrence of these motifs is coincidental. Manual inspection of the breakpoint regions and the junction fragment revealed a mirror repeat at the telomeric breakpoint. Such mirror repeats have the capacity to form noncanonical, three stranded structures referred to as H-DNA, being one of the non-B DNA structures [25]. H-DNA-forming sequences have previously been identified in regions that are prone to genomic rearrangements [25]–[28]. Interestingly, the pentanucleotide motif present in this mirror repeat is also seen on the reverse strand at the centromeric end of the deletion (Figure 3). We thus hypothesize that a double-stranded break (DSB) at the telomeric side triggered the deletion, followed by a DSB repair mechanism guided by the formation of a knot loop between the reverse complement of the pentanucleotide motif at the centromeric end (Figure 3).

The drastically reduced SRO contains 8 out of 25 CNCs identified in the initial SRO. Moreover, 4 out of 8 are conserved up to chicken, adding weight to an assumed functional role (Table 1). According to several miRNA databases the reduced SRO does not contain any miRNAs. We also investigated the regulatory potential of the deleted region using regulatory tracks. Based on the currently available data, the region is devoid of CpG islands, transcription start sites, conserved transcription factor binding sites, miRNA regulatory sites, VISTA enhancers, regulatory elements from OregAnno, *DNase*I hypersensitivity sites and CTCF binding sites. While the new SRO is devoid of known human genes, it does contain 4 human ESTs. Three are unspliced ESTs from two testis cDNA libraries, sharing a common telomeric end position (Figure 2). BLASTn analysis with EST AI204197 as query sequence retrieved 51 hits, including a significant alignment with *Capra hircus PISRT1* mRNA and *Mus musculus Pisrt1* partial mRNA sequence. *PISRT1* is one of the genes affected by the causal PIS deletion in goat. The PIS goat is the only known natural animal model for BPES associated with absence of horns (polledness) and intersexuality, caused by a regulatory 11.7 kb deletion located 280 kb upstream of goat *FOXL2*. It was shown that the deletion does not contain, but alters the transcription of at least three genes: *FOXL2*, the non-protein coding gene *PISRT1*, and *PFOXic* [14],[15]. Pailhoux et al. (2001) suggested that the PIS deletion harboured elements involved in long-range *cis*-regulation of goat *FOXL2* and *PISRT1*, as the expression of both genes is affected by the deletion [14]. This was further supported by our previous findings, revealing that the initial 126 kb SRO 5′ to *FOXL2* contains the PIS locus [10]. Here, the 7.4 kb deletion proved to contain the *PISRT1* orthologue, but not the PIS deletion (Figure 2). This suggests the existence of distinct interspecies *cis*-regulatory elements, which have similar effects when disrupted. Caprine *PISRT1* encodes a long non-coding transcript (ncRNA) of 1.5 kb that is highly expressed in adult testis [13]. A full-length cDNA of 758 bp was identified by 5′ RACE PCR starting from the known testis ESTs containing a polyadenylation site (Figure S1). These findings confirm its expression in human testis. In addition, no expression could be detected in fibroblasts, while a low *PISRT1* expression could be observed in KGN cells, indicating a co-expression of *PISRT1* and *FOXL2* in adult ovarian granulosa cells. These findings are consistent with expression profiles in goat and mice [13]. The latter is in line with a presumed regulatory function of *PISRT1*, requiring a tissue and cell-type specific expression.

Apart from copy number analyses, the remaining negative patients were specifically screened for sequence variants of CNCs within the initial SRO (Figure 2). To date, there are only a few human phenotypes found to be associated with sequence variations within *cis*-regulators [19],[20],[29],[30]. In this study, we identified 15 single nucleotide substitutions within CNCs or in flanking nucleotides and a 4-bp deletion mapping immediately upstream of CNC14 (Table S1). Only 3 nucleotide substitutions were found in BPES patients exclusively. However, no parents were available of these particular patients for segregation analysis (Table S1). Moreover, computational transcription factor binding site (TFBS) prediction on any of the wild type and variant CNCs did not support the creation or abolition of a TFBS.

**Figure 4:**
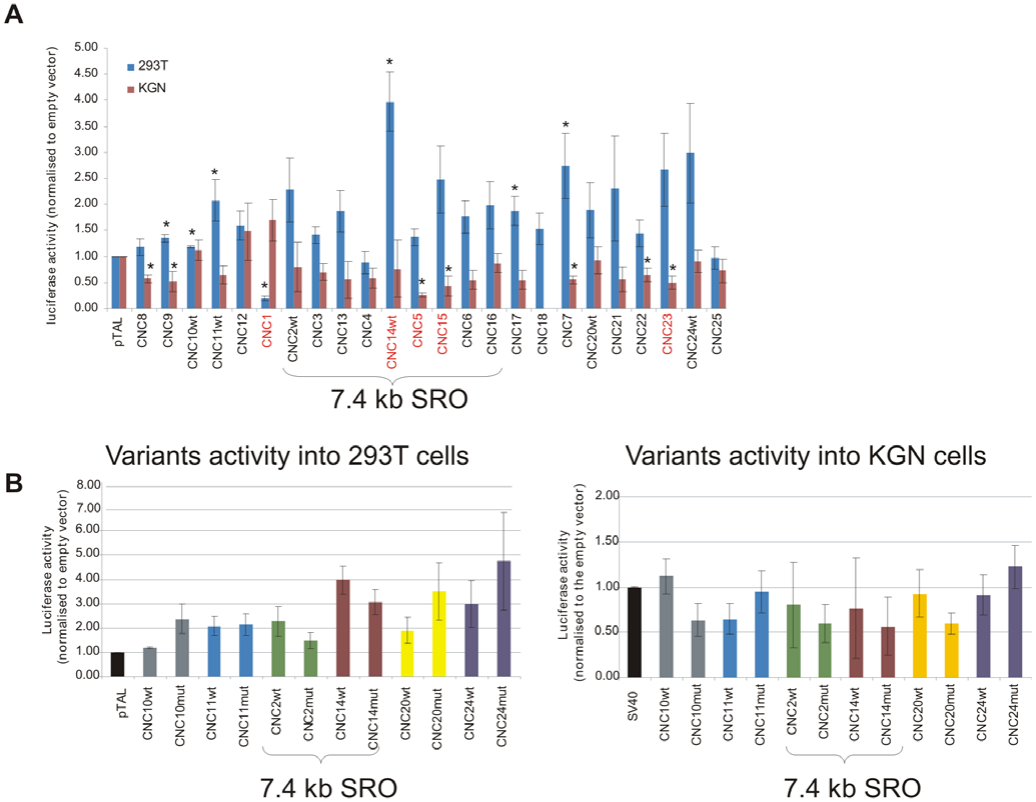
Regulatory activity of wild-type and variant CNCs in *FOXL2* expressing and non-expressing cells. The 25 identified CNCs and their putative pathogenic variants were cloned into a luciferase reporter vector and assayed for their regulatory role into expressing KGN and non-expressing 293T (human kidney cells) cells. (A) Bar chart showing the regulatory activity of each independent wild type CNC (CNCwt) in KGN (brown bars) and 293T (blue bars) relative to the basal activity of the empty vector (pTAL). Significant changes (one sample T-test, p value<0.05) are highlighted by an asterisk. CNCs with a >2 fold-change in activity between cell lines and a significant p value are shown in red. (B) Bar chart of wtCNCs and variant CNCs (CNCmut) regulatory activity in 293T and KGN cell lines respectively. Each CNC activity is first normalised to the basal activity of the empty vector and significant changes between wt and mut were then assessed by a two-samples T-test. No significant p values were found for any assayed construct (P value<0.05).

Although comparative sequence analysis has been proven to be a powerful approach to identify regulatory elements, experimental studies are required to confirm their role in gene regulation. The ability to modulate expression of a linked minimal promoter element in transient cell transfections is a widely exploited *in vitro* test of *cis*-regulatory potential [31]. Thus, for 24/25 CNC identified in the original SRO, *in vitro* luciferase assays were conducted (CNC19 could not be cloned). In both the KGN and 293T cell line, 29% (7/24) of the tested CNCs showed a significant difference in luciferase activity compared to the basal activity of the vector itself (T-test, P value<0.05) (Figure 4). Interestingly, cell-type specific regulatory potential could be observed among the constructs tested, three of which map within the 7.4 kb reduced SRO (CNC14, CNC5 and CNC15). This cell type specific regulatory activity supports that at least a fraction of the tested CNCs might be involved in the tissue-specific expression of *FOXL2*. We also addressed the putative functional impact of the identified nucleotide variants, but did not detect significant effects. A small quantitative and tissue-specific *cis*-regulatory effect of an individual CNC variation cannot be ruled out however. These results suggest that sequence variations within individual CNCs do not directly contribute to the molecular pathogenesis of BPES in our study.

As an additional experimental tool, 3C was conducted for a large region of 625 kb flanking the *FOXL2* gene. Using 3C, physical interactions between regulatory elements and their target genes can be demonstrated [32]. In the *FOXL2* expressing KGN cell line, the *FOXL2* core promoter containing *Eco*RI fragment 58 proved to come in close vicinity to *Eco*RI restriction fragments 109, 133 and 158, located 177, 283 and 360 kb upstream of *FOXL2* respectively (Figure S2). Moreover, an identical but lower interaction profile was detected in expressing fibroblast cells from a normal individual (F2) (Figure S2). These data demonstrate that in the nucleus of expressing cells, the promoter region of the *FOXL2* gene interacts with three long-distance *cis*-regulatory sequences.

**Figure 5:**
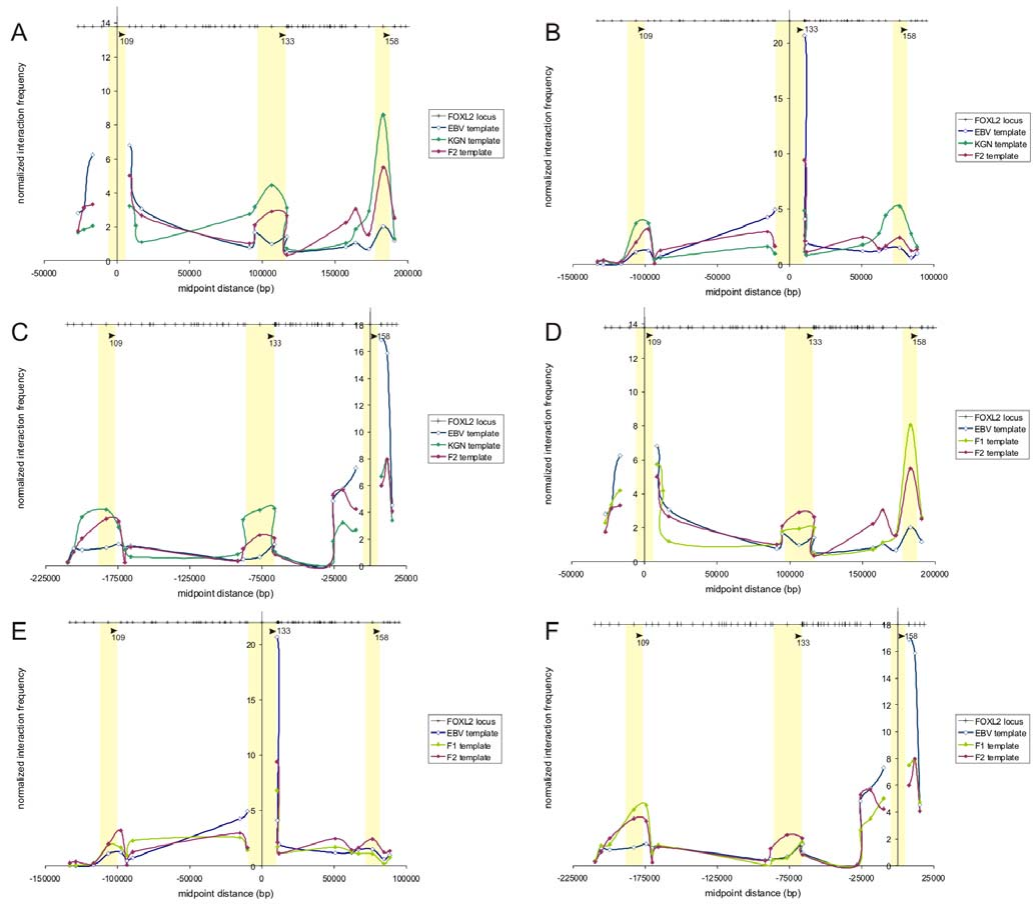
3C analysis of *FOXL2* region: mutual interactions between three regulatory sequences upstream of *FOXL2*. (A–C) In a first step 3C analysis of the *FOXL2* region demonstrated a close proximity of three evolutionarily conserved fragments 109, 133, and 158 with a fragment containing the *FOXL2* core promoter in expressing cells (KGN and control fibroblasts F2) (Figure 1, Figure 2, and Figure S2). Fragment 133 contains the 7.4 kb deletion. Second, to validate mutual interactions between these three regulatory fragments, 3C was performed in non-expressing EBV and expressing KGN and F2 cells with fragments 109 (A), 133 (B) and 158 (C) as anchor fragments respectively. The X-axis shows the genomic positions relative to the respective anchor fragments 109, 133 and 158 respectively; the Y-axis indicates normalized interaction frequencies measured by semi-quantitative PCR. At the Y-axis there are no peaks in interaction frequencies because an anchor fragment cannot interact with itself. Regions of interaction are highlighted with yellow rectangles. In expressing cells, all three distant fragments mutually interact and contact the *FOXL2* core promoter, assuming the intervening DNA loops out. Interaction frequencies between the *FOXL2* promoter and the regulatory sequences (represented in Figure S2) are significantly lower compared to interaction frequencies observed amongst the interacting fragments themselves (A–C). (D–F) The X-axis shows the genomic positions relative to the respective anchor fragments 109, 133, and 158 respectively; the Y-axis indicates normalized interaction frequencies measured by semi-quantitative PCR. At the Y-axis there are no peaks in interaction frequencies because an anchor fragment cannot interact with itself. Regions of interaction are highlighted with yellow rectangles. Experiments with anchor primers 109, 133, and 158 respectively (D–F), revealed interaction comparable to those in EBV cells in the deleted region. Moreover, Figure 5D and 5F show that in F1 cells, restriction fragments 109 and 158 maintain their mutual interaction in spite of absence of interaction with fragment 133. This demonstrates that retained mutual interactions and interactions between fragments 109 and 158 and the *FOXL2* core promoter are not sufficient for a normal cell-specific control of *FOXL2* expression.

To validate mutual interactions between these three regulatory regions, 3C was performed in EBV, KGN and F2 cells with fragments 109, 133 and 158 respectively as anchor fragments in a second step (Figure 5). It was found that in expressing cells, all three distant sequences mutually interact and contact the *FOXL2* core promoter, assuming that the intervening DNA loops out. Interestingly, fragment 133 contains the 7.4 kb fragment that is deleted in deletion D (Figure 1 and Figure 2; Table 1). To investigate the consequences of a heterozygous deletion of interacting fragment 133 on the interaction profile of the *FOXL2* locus, we analysed the mutual interactions of these three fragments in a fibroblast cell line F1, obtained from a BPES patient carrying an upstream deletion defining the initial SRO [10]. As a control, we used the fibroblast cell line F2. Interactions of the promoter with the two elements 109 and 158 that are not located within the deletion are not reduced. Thus, this suggests that even on the deleted chromosome these elements can interact with the *FOXL2* promotor despite the absence of fragment 133. Furthermore, fragments 109 and 158 appear to mutually interact even in the absence of 133. The upstream deletion disrupts fragment 133 within the reduced SRO, and causes a BPES phenotype. The latter might lead to the conclusion that the retained interactions between fragments 109 and 158 and the *FOXL2* core promoter are not sufficient to correctly regulate *FOXL2* transcription in the adult expressing cell system studied here. Moreover, it implies that the interactions between the *cis*-regulatory element(s) located in fragment 133 and the *FOXL2* core promoter are essential for this.

### General conclusions and perspectives

We identified a *de novo* distant 7.4-kb deletion that is causally related to BPES. To our knowledge, this is the smallest fully characterized distant deletion implicated in the causation of a human genetic condition (Table S2). This deletion disrupts a long ncRNA *PISRT1* and 8 CNCs, 4 of which are conserved up to chicken. Functional assays suggest a *cis*-regulatory and tissue-specific potential of 3 of them. The biological relevance of these findings was corroborated by the 3C study of a normal and aberrant *FOXL2* locus in expressing adult cellular systems respectively, demonstrating a close proximity of the 7.4 kb deleted fragment and two other conserved regions with the *FOXL2* core promoter, and the necessity of the integrity of the regulatory domain for correct *FOXL2* expression.

Altogether, we identified and characterized a novel tissue-specific *cis*-regulatory domain of *FOXL2* expression. As we demonstrated the consequences of its disruption, our findings impact mutation screening of strictly regulated developmental and other disease genes. Specifically, our study emphasizes the need for high-resolution copy number screening of their *cis*-regulatory domains. Genome-wide tools such as oligonucleotide or SNP arrays and next-generation sequencing will play a prominent role in this. In addition, a well-selected patient population is another requirement, as illustrated here: (1) we only included patients with a diagnosis of BPES, a clinically distinguishable but rare disorder, and (2) they all underwent a uniform pre-screening excluding intragenic *FOXL2* mutations and gene deletions.

Sequence variations within individual CNCs did not contribute to the molecular pathogenesis of BPES in our study. This can be explained by the fact that sequence changes within individual CNCs might result in a more subtle, different or even normal phenotype, as the *cis*-regulatory elements they represent might act in a tissue-specific and quantitative manner [5],[6],[19],[33]. The most striking example of the latter is the differential phenotype caused by point mutations in *SHH* and in its limb-specific enhancer ZRS of *SHH*, resulting in holoprosencephaly type III (HPE3) (OMIM 142945) and PPD respectively [20],[34].

Other mechanisms may explain the phenotype in the remaining 53 molecularly undefined BPES patients. Although there is no clear evidence for locus heterogeneity in BPES, mutations in other disease genes apart from *FOXL2* cannot be excluded in some of the remaining molecularly unresolved cases. Another possibility is the occurrence of regulatory variants within the untranslated regions (UTRs) or the core promoter. A number of non-pathogenic sequence variants have been reported in the *FOXL2* putative core promoter and untranslated regions (UTRs) up to now. However, a single basepair insertion in the *FOXL2* 3′UTR was found to co-segregate with BPES in a large Chinese type II BPES family, and was shown to be located in an AU rich repeat [35]. No functional studies were provided however to unequivocally prove a relationship between the insertion and the phenotype in this family. Interestingly, in the *FOXP3* gene (NM_014009), encoding another forkhead transcription factor, a presumed disease-causing sequence change was found in the 3′UTR within the poly(A) signal, in affected members of a five-generation family with X-linked immune dysfunction, polyendocrinopathy, enteropathy (IPEX) (MIM 304790) [36]. The occurrence of interesting pathogenic or modifying variants in 3′UTRs is in line with their important role in the regulation of gene expression at both pre-mRNA, mature mRNA and post-transcriptional level through *cis*-acting elements that interact with a variety of *trans*-acting factors [37]. This is highlighted by their many conserved sequence motifs, including microRNA (miRNA) targets [37]. It cannot be ruled out that changes in post-transcriptional regulation by altered miRNA targeting may result in BPES. A unique example of a variant that alters the gene expression level by modifying miRNA targeting activity is a 3′UTR SNP in human *SLITRK1* (NM_052910), which is implicated in Tourette syndrome (MIM 137580) [38].

Finally, this study considerably adds to the importance of an intact tissue-specific *cis*-regulatory domain in this and other developmental disorders. This impacts upon the concept of mutation screening of developmental disease in particular, and of human genetic disease in general. In the future, online databases such as Decipher and the Database of Genomic Variants which collect information on copy number changes, might help to interpret copy number changes affecting putative regulatory regions that might lead to disease [39],[40].

## Materials and Methods

### Patients

Genomic DNA (gDNA) from 57 consenting BPES patients without intragenic mutation or copy number change of the *FOXL2* coding region was used in this study. Criteria described previously were used to accept a diagnosis of BPES [9]. The study was conducted following the tenets of Helsinki and was approved by the local Ethics Committee of the Ghent University Hospital.

### Microsatellite analysis

In order to detect hemizygous regions outside *FOXL2*, microsatellite analysis was performed as described previously [10]. Microsatellite analysis was conducted for 19 molecularly unresolved patients for whom parental DNA was available.

### ArrayCGH

In order to detect copy number changes outside the transcription unit of *FOXL2*, a new purpose-built bacterial artificial chromosome (BAC) array, consisting of 132 unique genomic clones covering a region of 3 Mb around *FOXL2* and 95 control BACs (3 on each chromosome and 26 on the X chromosome), was designed in-house as previously described [41],[42]. In total, 500 ng of DNA was labelled by a random prime labelling system (BioPrime ArrayCGH genomic labelling system, Invitrogen) using Cy3 and Cy5 labelled dCTPs (Amersham Biosciences). Hybridizations were performed automatically using the HS400 hybridization station (Tecan) for 21 molecularly unresolved patients, of which 13 were previously screened by microsatellite analysis. The scan images were processed with Imagene software (Biodiscovery) and further analysed with arrayCGHbase [43].

### Real-time quantitative PCR (qPCR) in the *FOXL2* region (qPCR-3q23)

Quantitative qPCR (qPCR-3q23) was performed as described for a second group of patients as an alternative to arrayCGH, in order to detect copy number changes encompassing the initial SRO [44]. First, 3 qPCR amplicons located within the SRO 5′ to *FOXL2* were designed and used to identify possible extragenic deletions overlapping the SRO in 24 molecularly unresolved patients, not previously screened by array CGH. Second, 10 additional in-house designed amplicons were used to further delineate 3 new extragenic deletions. All 13 amplicons were designed *in silico* as described (primer sequences available upon request) [44]. qPCR was carried out using the qPCR Core kit for SYBR Green I (Eurogentec) on the LightCycler 480 (Roche). Calculation of the gene copy number was performed with qBase software [45]. Two reference genes, *ZNF80* (NM_007136) and *GPR15* (NM_005290), were used for normalization of the relative quantities.

### Comparative sequence analysis (identification of CNCs)

A comparative analysis of the SRO region (delineated by SNP rs10935309 and rs4894405) was performed by pairwise comparison of the human and mouse genomes. More specifically, the GALA genome browser implemented with hg16 build was used to identify all non-coding sequences of ≥100 bp and sharing ≥70% identity with the mouse [46]. The analysis resulted in the identification of 25 CNCs that are reproducibly mapped when implementing the hg17 build.

Subsequently, using the multiZ alignment track in the UCSC genome browser, the conservation of all identified CNCs was examined in the genomes of placental mammals, chicken and pufferfish. In addition, the overlap of all the identified CNCs with previously reported PhastCons sequences was evaluated using the PhastCons conservation in the UCSC Genome Browser [47],[48].

### qPCR for CNCs in the SRO (qPCR-CNC)

In order to detect subtle copy number changes within or nearby the identified CNCs specifically, 36 qPCR amplicons were designed within the initial SRO: 19/36 map within CNCs (no successful assays were obtained for CNC10, 14, 15, 21, 23 and 25) while the 15 additional assays map within some long flanking regions. The latter amplicons were designed following CNC copy-number analysis in order to increase the screen resolution or to verify the mapping of putative copy-number variants. SYBR Green I qPCR-CNC was performed in 53 selected patients as described [49]. All amplicons were designed *in silico* using PrimerExpress (Applied Biosystems) (primers available upon request) and validated as described [49].

### Deletion-junction PCR, sequencing, and *in silico* analysis of the breakpoint regions

For the patient with deletion D long-range PCR was performed using the qPCR primers delineating the deletion. For long-range PCR the iProof high-fidelity PCR kit (Biorad) was used according to the manufacturer's instructions.

In order to determine the junctions at base pair resolution, direct sequencing was performed on the 5 kb product using 8 internal sequencing primers (available upon request) (ABI 3730xl Applied Biosystems). We used several web-based tools to unravel the mechanism by which the 7.4 kb deletion occurred. Genomic sequences of several sizes and centered on the breakpoints were obtained from the UCSC genome browser. First, CLUSTALW was used to align the junction sequence (70 bp) with the reference genomic sequence from both the proximal and the distal breakpoint region [50]. Second, BLAST2 was run under default conditions to perform a pairwise sequence comparison of the 2 kb proximal and distal breakpoint regions [51]. Third, several programs (RepeatMasker, Mreps, Palindrome, and Censor) were employed to screen for repetitive elements/structures, low-complexity sequences, tandem and palindromic inverted repeats [52]–[54]. For sequence analysis with RepeatMasker and Censor we used the 2 kb breakpoint regions and for analysis with Mreps and Palindrome 300 bp regions. In addition, the fractional GC content of the breakpoint regions was calculated using GEECEE.

DNA Pattern Find was applied to locate specific sequence motifs within the 70 bp breakpoint regions and the junction fragment [55]. The investigated specific sequences are known to be implicated in DNA rearrangements elsewhere [56].

### *In silico* analysis of the reduced SRO

Several tracks within the UCSC genome browser (Genes and Gene Prediction Tracks, mRNA and EST Tracks and Regulation Tracks) were used to screen the reduced SRO (chr3:140,431,841-140,439,199). In addition, the Ensembl regulatory features track was used to gain information about possible *DNase*I hypersensitivity sites and CCCTC-binding factor (CTCF) binding sites. Several RNA databases (RNAdb, miRDB, miRNAMap, miRBase and NONCODE v2.0) were consulted in order to extract possible non-coding RNA sequences [57]–[61]. Finally, BLASTn was run under default conditions to define the human orthologue of caprine *PISRT1* (AF404302) within this reduced SRO. In order to define the location of the human 7.4 kb deletion with respect to the deletion in the PIS goat, BLAST2 was performed for goat BAC 376H9 and a 100 kb extract from human chromosome 3 NT_005612.15 containing the reduced SRO (45.400.000–45.500.000).

### Expression analysis and 5′ RACE of human *PISRT1*

Relative *PISRT1* expression levels were determined in several human cell lines/tissues using real-time quantitative RT-PCR with newly designed primers (available upon request). Primers were designed as described [44]. cDNA prepared from fibroblasts from a control individual and from human granulosa KGN cells (Riken Institute) and cDNA from testis (human testis Marathon-Ready cDNA, Clontech) were used for *PISRT1* expression analysis.. RNA was isolated from fibroblasts and KGN cells as described (RNeasy, Qiagen), and treated with RNase-free DNAse (Promega), followed by cDNA synthesis as described (iScript cDNA synthesis kit, Bio-Rad); qPCR was carried out using the qPCR Core kit for SYBR Green I (Eurogentec) on the LightCycler 480 (Roche) as described above. *PISRT1* expression levels were normalized using 3 housekeeping genes (*HPRT1*, *GAPDH* and *YWHAZ*) (NM_000194, NM_002046 and NM_145690). The obtained data were analyzed using qBase plus [45].

To characterize the full-length human *PISRT1* transcript, 5′ rapid amplification of the cDNA ends (5′ RACE, Clontech) was performed according to the manufacturer's protocol, using the Advantage cDNA PCR Kit and human testis Marathon-Ready cDNA (Clontech) as a template (primers available upon request). For our novel human *PISRT1* transcript, an accession number was requested at the GenBank (accession number FJ617010).

### Sequencing of CNCs

Primers surrounding each of the 25 CNCs (±50 bp of the core CNC) were designed with Primer3 (primers available upon request) [62]. A specific amplicon could be obtained for 24/25 CNCs, except for CNC19. Sequence analysis of 24 CNCs was performed in 32 molecularly unresolved patients. In a second step, targeted sequencing of CNCs mapping within the reduced SRO defined by the 7.4 kb deletion, was performed in the remaining 21 patients.

Sequence analysis of the first set of patients was performed with RedTaq (Jumpstart kit, Sigma) under standard touchdown PCR conditions. For the second set of patients new amplicons were designed for closely mapping CNCs instead of single CNC analysis. Thus, CNC5, 15, 6, 16, 4 and 14 were pooled as follows: CNC5-15 (amplicon size: 573 bp), CNC6-16 (amplicon size: 962 bp) and CNC4-14 (amplicon size: 1140 bp). In this case, PCR amplification was carried out with the iProof High-Fidelity DNA polymerase (BioRad) as indicated by the manufacturer. Each amplicon was directly sequenced in forward and reverse orientation using an ABI 3130 analyser (Applied Biosystems). To align and identify nucleotide variants the Sequencher software (Gene Codes Corporation) was used. Multispecies alignments extracted from the UCSC Genome browser were used to evaluate the conserved nature of nucleotides presenting variants. Computational transcription factor binding site predictions were performed with the MATCH interface of the TRANSFAC database [63],[64].

### Luciferase constructs and assays

*In vitro* luciferase assays were performed in *FOXL2* expressing KGN cells, and non-expressing 293T cells (human kidney cells, ATCC). Wild-type (WT) and variant CNCs were directly PCR amplified from normal and affected genomic DNA respectively, with the same sets of primers and PCR conditions used for CNC sequencing, except for CNC1. For CNC1 new primers were designed as described above based on a recent conservation pattern survey. The new CNC1 amplicon adds approximately 260 bp to the original one and covers the full conserved alignment that can be observed in UCSC and that overlaps with an extremely conserved sequence with highly regulatory potential [65]. Two types of luciferase constructs were produced: (1) *pTAL-Luc CNC constructs*, for which each PCR product was cloned into the TOPO-TA PCR II vector after amplification (Invitrogen); colonies with insert in reverse orientation (i.e. 3′-5′) were specifically selected and sequenced. Subsequent subcloning into the pTAL-Luc vector (Clontech) expressing the firefly luciferase was achieved by *Sac*I-*Xho*I digestion of both the TOPO-CNC constructs and pTAL-Luc vector (Clontech). The amplicon encompassing CNC1 contained internal *Sac*I and *Xho*I, and was subcloned using *Spe*I-*Bgl*II restriction sites. The fragment was subsequently cloned into a modified pTAL-Luc vector containing part of the multiple cloning site of TOPO-TA II. (2) *pTAL-SV40 CNC constructs*, for which the pTAL-Luc backbone was digested with *Bgl*II and *Hind*III in order to remove the minimal TATA-like promoter and replace it by a SV40 promoter. Subsequently, all pTAL-Luc CNCs were digested with *Sac*I-*Xho*I (*Spe*I-*Bgl*II for CNC1) and subcloned into a pTAL-SV40 (Promega) digested with similar enzymes. In both approaches, reverse-orientated CNC constructs were obtained. We specifically decided to investigate the regulatory potential of CNCs in their native orientation with respect to *FOXL2*.

#### Luciferase Assays in 293T and KGN

The assay was performed as previously described [31]. For KGN, transfections were performed with minor modifications; briefly, 1×10^5^ cells/well were grown into 24 wells and transiently transfected with 0.5 µg of each pTAL-SV40 CNCs construct along with 100 ng of *renilla* control plasmid (pRL-SV40).

For both cell lines, each construct was assayed in triplicate in three independent experiments. Firefly and *renilla* luciferase activities were measured using the Dual-Glo Luciferase Assay System (Promega) and a microplate luminometer (VICTOR^3^, PerkinElmer). We determined the luciferase activity driven by each construct by first measuring the firefly to *renilla* luciferase ratio for each transfection. In a second step, the signal was normalized to the control ratio (pTAL-Luc/pRL-SV40 or pTAL-SV40/pRL-SV40) included on each plate. Standard deviations were calculated for each construct.

In a next step, the putative influence of the variant on the level of transcription was expressed as the fold change in luciferase activity over the basal activity of the luciferase with the WT version of the respective CNC. P-values were calculated by 2-sample T-test. Significant differences, i.e. P<0.05, were indicated by an asterisk.

### Chromosome conformation capture (3C)

#### BAC selection and control library preparation

To create a standard for normalization of relative PCR efficiencies, a control template for the human *FOXL2* locus and gene desert regions (ENCODE region ENr313) was generated using a set of minimally overlapping bacterial artificial chromosome (BAC) clones [66],[67]. The following five BAC clones were used for the *FOXL2* locus: RP11-579O13, RP11-259D13, RP11-1129G19, RP11-111F8 and RP11-203B18. A set of four BAC clones was selected to cover the 0.5-Mb gene desert region and include RP11-197K24, RP11-609A13, RP11-454G21 and CTD-2133M23. These BAC clones were obtained from the Children's Hospital Oakland Research Institute (CHORI) and Invitrogen. BAC preparations were quantified by real-time quantitative PCR with SYBR Green I using universal primers that amplify part of the BAC vector backbone. Subsequently, BAC DNA was mixed in equimolar ratios, digested with *Eco*RI and randomly ligated, to obtain a collection of all possible ligation products in equimolar amounts.

#### Cell lines and culture conditions

The KGN cell line was grown as described [68]. A control EBV cell line and fibroblasts were grown in standard conditions. The EBV cell line was derived from EBV-transformed B-lymphocytes of a healthy control. Template F1 was generated from fibroblasts from a BPES patient, carrying a deletion outside the transcription unit of *FOXL2* described by us [10]. A fibroblast cell line derived from a normal individual was used to create the F2 template. *FOXL2* expression in the KGN, F1 and F2 cell lines was verified by real-time quantitative PCR (primers available upon request).

#### 3C assay and PCR analysis of the ligation products

This was essentially performed as described [32],[66] [67]. Primers were designed to flank *Eco*RI sites with an orientation that allows amplification of potential ligated sequences. The 5′ side of each restriction fragment was used to design primers unless this coincided with repetitive DNA sequences (primers available upon request). The sizes of the predicted PCR products varied from 172 to 388 bp. The linear range of amplification was determined by using serial dilutions of the control template and all experimental templates for four different primer pairs. PCRs were conducted in 25 µl under the same cycling conditions, followed by agarose electrophoresis quantified on a Kodak Image Station 440 CF (Kodak).

#### Experimental controls

Several experimental controls were included to rule out potential artefacts [66]. First, a BAC control template was generated to normalize for differences in primer efficiency. This control template contains equimolar amounts of all possible ligation products of the region of interest and the gene desert regions. Second, the level of background random collisions was assessed by determination of interactions between sites separated by increasing genomic distances, ranging from 0 to 130 kb both 3′ and 5′ of *FOXL2*. In all cell lines, highest interaction frequencies were found with neighbouring fragments upstream and downstream of the *FOXL2* core promoter, reflecting non-functional random collisions from adjacent restriction fragments. Moreover, interaction frequencies gradually decreased with fragments located further away on the DNA template. Finally, to allow direct quantitative comparison of interaction frequencies determined in all cell lines, interaction frequencies were normalized using a set of 18 interaction frequencies detected in gene desert regions (ENCODE region ENr313), assuming that this region has a similar conformation in all cell types. The average log ratio of these interaction frequencies was calculated in all cell types to determine the average fold difference in interaction frequencies between the EBV template and the KGN, F1 or F2 template.

### Web resources

Ensembl Genome Browser, http://www.ensembl.org/index.html

GEECEE, http://mobyle.pasteur.fr/cgi-bin/MobylePortal/portal.py?form=geecee

GenBank (MapViewer), http://www.ncbi.nlm.nih.gov/mapview/static/MVstart.html

Online Mendelian Inheritance in Man (OMIM), http://www.ncbi.nlm.nih.gov/omim/

Palindrome, http://bioweb.pasteur.fr/seqanal/interfaces/palindrome.html

Repeatmasker, http://www.repeatmasker.org

UCSC Genome browser, http://genome.ucsc.edu/

## Supporting Information

Figure S1 Alignment of *PISRT1* homologues based on BLAST searches and 5′ RACE. Goat mRNA sequence AF404302 was used for BLASTN searches against human and mouse genomes. The homologous regions were localized on human contig NT_005612.15 at position 45445646–45447509 and on the mouse contig NT_039476.7 at position 18278112–18279127. These retrieved sequences, the human EST AW268472 and the canine EST CO633486.1 were aligned with ClustalW. Identification of the full-length human transcript was performed by 5′ RACE starting from testis-specific EST AW268472 using a testis cDNA library. The 2 gene specific primers used for 5′ RACE are indicated in red. The 5′ end of the full-length transcript is marked by a vertical black line. The reference number of the novel human PISRT1 was requested and retrieved at Genbank (FJ617010).
(7.21 MB TIF)

Figure S2 3C analysis of the human *FOXL2* locus in EBV, KGN and F2 cells. Schematic representation of the *FOXL2* locus. In the top line, genes located in this region are depicted by coloured boxes. The second line indicates the SROs of the downstream deletion (dashed line on the left) and the initial SRO of upstream deletions (red dashed line on the right respectively). Hatch marks on the third line represent midpoint distances of the *Eco*RI restriction fragments to anchor fragment 58. Arrowheads correspond with the location of the respective primers. The positions of the three known translocation breakpoints at 3q23 in BPES and of the orthologue of the PIS deletion are indicated by vertical arrows at the top. At the bottom, dot plot of 3C analysis representing interaction frequencies between the *Eco*RI fragment overlapping the *FOXL2* promoter (fragment 58) and restriction fragments throughout the *FOXL2* locus in non-expressing EBV cells, and expressing adult granulosa KGN and fibroblast cells F2. The X-axis shows the genomic position relative to anchor fragment 58; the Y-axis indicates normalized interaction frequencies measured by semi-quantitative PCR. Regions of interaction are highlighted with yellow rectangles. In the KGN cell line, the fragment containing (58) the *FOXL2* core promoter is shown to come in close vicinity to EcoRI restriction fragments 109, 133, and 158, located 177, 283, and 360 kb upstream of *FOXL2* respectively. The fold differences (average ratio of normalised interaction frequencies) of these interactions are 8, 11, and 39 respectively. An identical but lower interaction profile is seen in expressing fibroblast cells from a normal individual (F2). *Eco*RI fragments 109, 133 and 158 all correspond to evolutionarily conserved elements described by Crisponi et al. 2004 (see also Figure 1, Figure 2, and Table 1). Fragment 133 contains the reduced SRO of 7.4 kb and thus the *PISRT1* transcript. Altogether, these 3C data demonstrates that in the nucleus of expressing cells, the promoter region of the *FOXL2* gene comes in close vicinity to three distant cis-regulatory sequences that correspond to conserved sequence blocks.
(8.46 MB TIF)

Table S1 Variants identified by sequence analysis of CNCs.
(0.04 MB DOC)

Table S2 Reported extragenic deletions in human genetic disorders.
(0.11 MB DOC)
