# Supplementary figures and images for: Disease-Causing 7.4 kb Cis-Regulatory Deletion Disrupting Conserved Non-Coding Sequences and Their Interaction with the FOXL2 Promotor: Implications for Mutation Screening
Source: PLoS Genet. 2009 Jun 19;5(6):e1000522. doi: 10.1371/journal.pgen.1000522 (PMC2689649; doi:10.1371/journal.pgen.1000522)

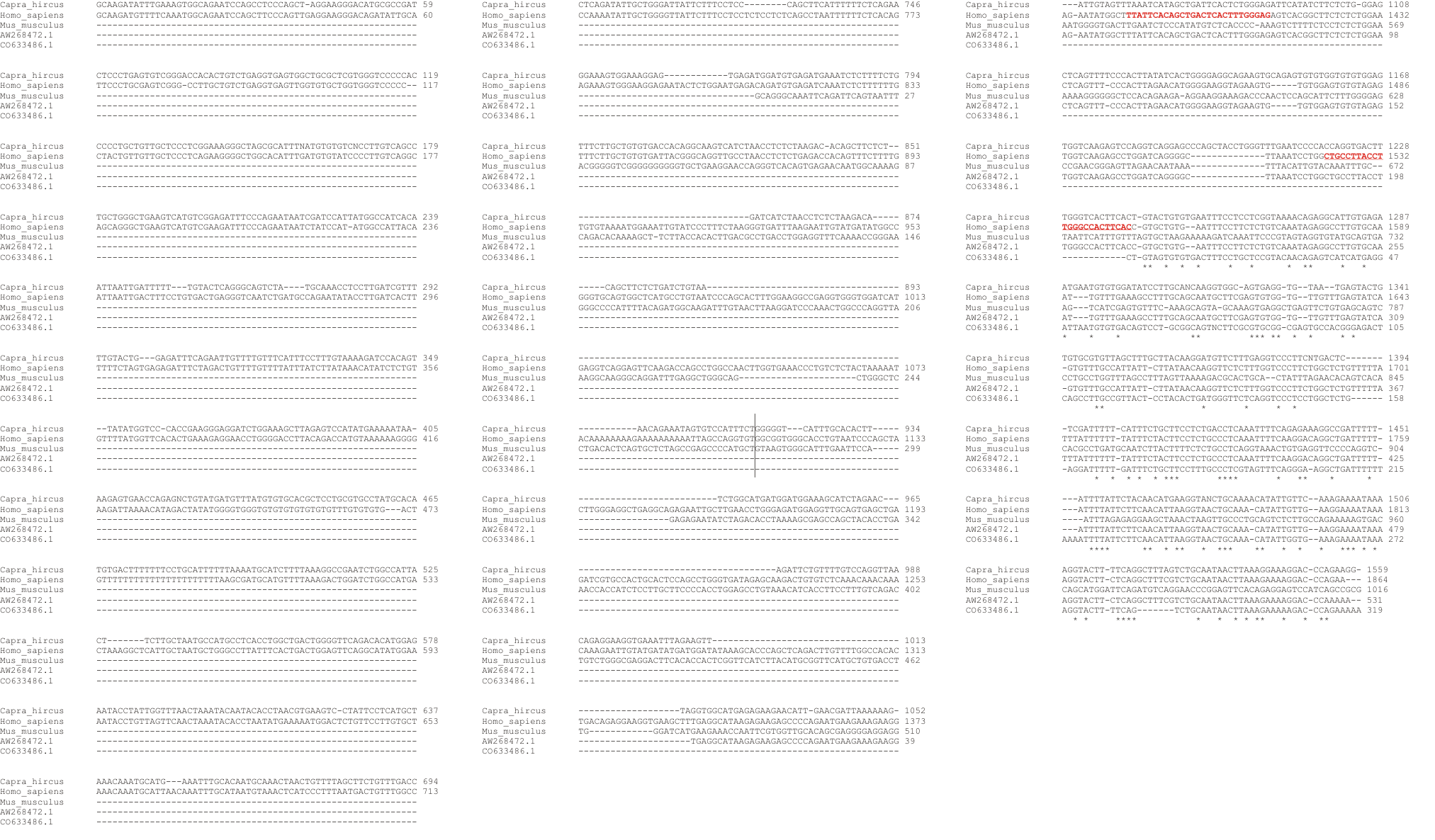

Supplement: Figure S1 — Alignment of PISRT1 homologues based on BLAST searches and 5′ RACE. Goat mRNA sequence AF404302 was used for BLASTN searches against human and mouse genomes. The homologous regions were localized on human contig NT_005612.15 at position 45445646–45447509 and on the mouse contig NT_039476.7 at position 18278112–18279127. These retrieved sequences, the human EST AW268472 and the canine EST CO633486.1 were aligned with ClustalW. Identification of the full-length human transcript was performed by 5′ RACE starting from testis-specific EST AW268472 using a testis cDNA library. The 2 gene specific primers used for 5′ RACE are indicated in red. The 5′ end of the full-length transcript is marked by a vertical black line. The reference number of the novel human PISRT1 was requested and retrieved at Genbank (FJ617010). (7.21 MB TIF) [file pgen.1000522.s001.tif]

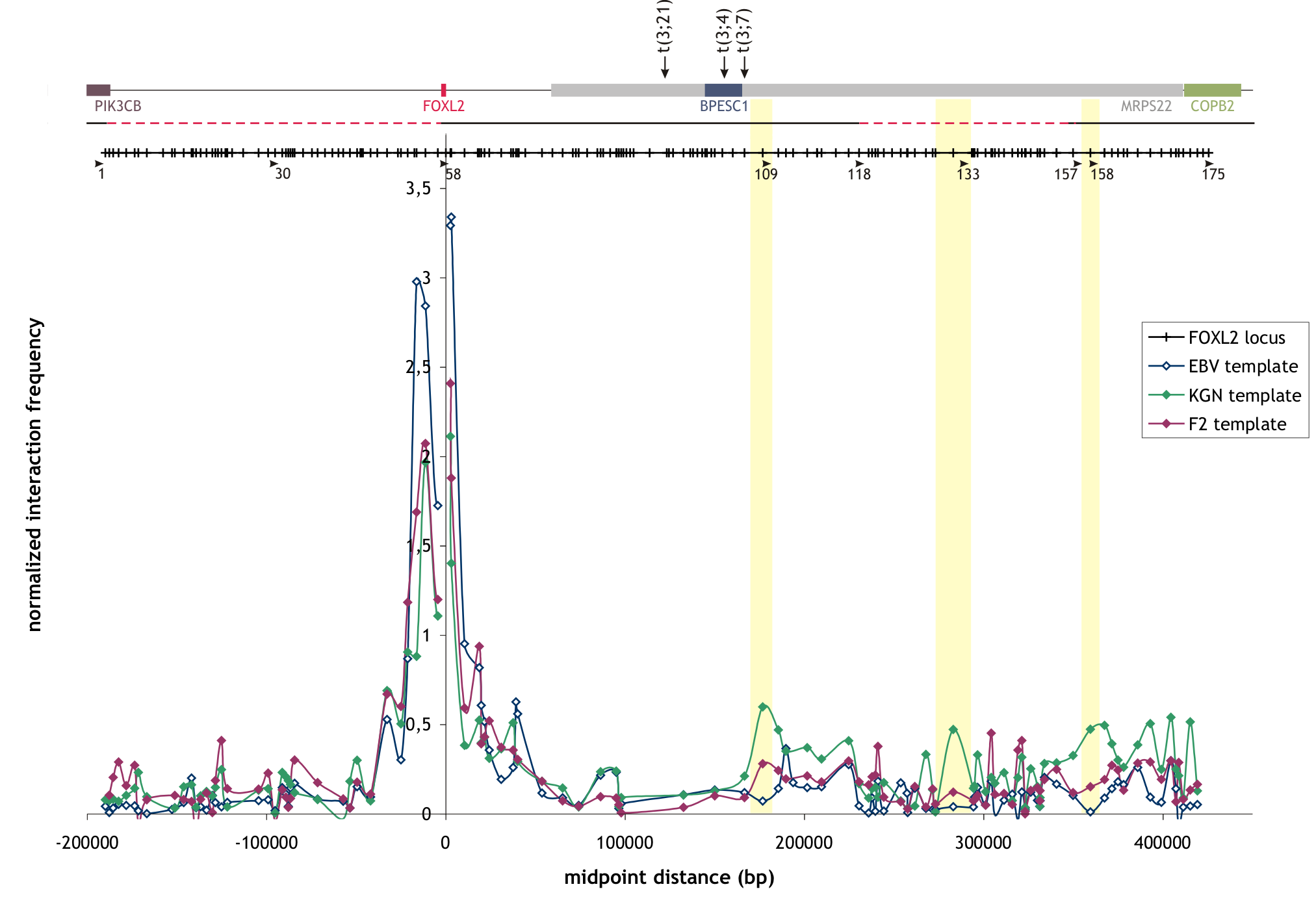

Supplement: Figure S2 — 3C analysis of the human FOXL2 locus in EBV, KGN and F2 cells. Schematic representation of the FOXL2 locus. In the top line, genes located in this region are depicted by coloured boxes. The second line indicates the SROs of the downstream deletion (dashed line on the left) and the initial SRO of upstream deletions (red dashed line on the right respectively). Hatch marks on the third line represent midpoint distances of the EcoRI restriction fragments to anchor fragment 58. Arrowheads correspond with the location of the respective primers. The positions of the three known translocation breakpoints at 3q23 in BPES and of the orthologue of the PIS deletion are indicated by vertical arrows at the top. At the bottom, dot plot of 3C analysis representing interaction frequencies between the EcoRI fragment overlapping the FOXL2 promoter (fragment 58) and restriction fragments throughout the FOXL2 locus in non-expressing EBV cells, and expressing adult granulosa KGN and fibroblast cells F2. The X-axis shows the genomic position relative to anchor fragment 58; the Y-axis indicates normalized interaction frequencies measured by semi-quantitative PCR. Regions of interaction are highlighted with yellow rectangles. In the KGN cell line, the fragment containing (58) the FOXL2 core promoter is shown to come in close vicinity to EcoRI restriction fragments 109, 133, and 158, located 177, 283, and 360 kb upstream of FOXL2 respectively. The fold differences (average ratio of normalised interaction frequencies) of these interactions are 8, 11, and 39 respectively. An identical but lower interaction profile is seen in expressing fibroblast cells from a normal individual (F2). EcoRI fragments 109, 133 and 158 all correspond to evolutionarily conserved elements described by Crisponi et al. 2004 (see also Figure 1, Figure 2, and Table 1). Fragment 133 contains the reduced SRO of 7.4 kb and thus the PISRT1 transcript. Altogether, these 3C data demonstrates that in the nucleus of expressing [file pgen.1000522.s002.tif]
