# Supplementary material for: Disease-Causing 7.4 kb Cis-Regulatory Deletion Disrupting Conserved Non-Coding Sequences and Their Interaction with the FOXL2 Promotor: Implications for Mutation Screening
Source: PLoS Genet. 2009 Jun 19;5(6):e1000522. doi: 10.1371/journal.pgen.1000522 (PMC2689649; doi:10.1371/journal.pgen.1000522)
Supplement: Table S1 — Variants identified by sequence analysis of CNCs. (0.04 MB DOC) [file pgen.1000522.s003.doc]

**Table S1. Variants identified by sequence analysis of CNCs.**

|  | **CNC** | **Variant position ^a^** | **Variant ^b^** | **Number of patients exhibiting the variant** | **Observed frequency ^c^** | **Known frequency ^d^** |
| --- | --- | --- | --- | --- | --- | --- |
| Known SNPs | CNC10 | +139 | T/A | 1 | 0.009 (1/106) | 0.00 |
|  | CNC11 | +195 | C/T | 27 | 0.29 (31/106) | 0.39 |
|  | CNC18 | +126 | C/T | 3 | 0.038 (4/106) | 0.033 |
|  | CNC18 | +156 | A/C | 22 | 0.25 (26/106) | n.i. |
|  | CNC24 | +89 | C/T | 3 | 0.038 (4/106) | 0.083 |
| Non-causative SNPs | CNC3 | +68 | C/T | 7 | 0.046 (7/152^*^) | n.k. |
|  | CNC4-14 | +158 | C/T | 3 | 0.02 (3/152^*^) | n.k. |
|  | CNC4-14 | +363 | T/C | 3 | 0.02 (3/152^*^) | n.k. |
|  | CNC8 | +369 | C/T | 9 | 0.09 (10/106) | n.k. |
|  | CNC18 | +109 | A/G | 2 | 0.019 (2/106) | n.k. |
|  | CNC22 | +88 | A/G | 5 | 0.057 (6/106) | n.k. |
|  | CNC25 | +141 | A/G | 5 | 0.057 (6/106) | n.k. |
| Putative causative SNPs^e^ | CNC2 | +134 | T/C | 1 | 0.009 (1/106) | n.k. |
|  | CNC11 | +214 | T/C | 1 | 0.009 (1/106) | n.k. |
|  | CNC20 | +190 | C/T | 1 | 0.009 (1/106) | n.k. |

^a^ Position of the variant nucleotide according to the forward primer used for the sequencing

^b^ The reference genotype is not discussed, only the observed variant alleles are described (T/A means, reference genotype is T/T, etc.)

^c^ Number of variants/number of sequenced chromosomes, ^*^Including 21 additional patients and 2 parents specifically sequenced for CNCs within the 7.4 kb SRO region

^d^ Frequency reported for HapMap European population in the NCBI SNP database – dbSNP BUILD 129 (n.i.; not indicated, n.k.; not known)

^e^ Putative causative SNPs are those found exclusively in BPES patients.
