# Supplementary material for: Disease-Causing 7.4 kb Cis-Regulatory Deletion Disrupting Conserved Non-Coding Sequences and Their Interaction with the FOXL2 Promotor: Implications for Mutation Screening
Source: PLoS Genet. 2009 Jun 19;5(6):e1000522. doi: 10.1371/journal.pgen.1000522 (PMC2689649; doi:10.1371/journal.pgen.1000522)
Supplement: Table S2 — Reported extragenic deletions in human genetic disorders. (0.11 MB DOC) [file pgen.1000522.s004.doc]

**Table S2. Reported extragenic deletions in human genetic disorders.**

| **Disease** | **Locus** | **Gene** | **Distance of the deletion**  **from the gene (in kilobases);**  **orientation with respect to target gene** | **Size of the deletion (in kilobases)** | **References** |
| --- | --- | --- | --- | --- | --- |
| Aniridia, type II | 11p13 | *PAX6* | 22.1 kb; 3’  11.6 kb; 3’ | 975 kb deletion  1,105 kb deletion | [1] |
| α-thalassemia | 16p13.3 | *HBA2* | 0.35 kb; 3’  30-50 kb; 5’ | 18.4 kb, including *HBA1*, *HBAQ1* and part of *LUC7L*  also multiple overlapping deletions | [2]; [3]; [4] |
| Blepharophimosis syndrome (BPES) | 3q22.3 | *FOXL2* | 101-231 kb; 5’  28.7 kb; 3’ | 4 different microdeletions (126 kb to 1.9 Mb)  188 kb microdeletion | [5] |
| Campomelic dysplasia | 17q24.3 | *SOX9* | 380 kb; 5’ | 1.5 Mb microdeletion | [6] |
| Pierre Robin sequence (PRS) | 17q24.3 | *SOX9* | 1.38 Mb; 5’  1.58 Mb; 5’  1.56 Mb; 3’ | 75 kb microdeletion  >319 kb microdeletion  36 kb microdeletion | [7] |
| Glaucoma / iridogoniodysgenesis / Axenfield-Rieger syndrome | 6p25 | *FOXC1* | 1200 kb; 5’ |  | [8] |
| Gonadal dysgenesis with XY sex reversal | Yp11.31 | *SRY* | 1.7 kb; 5’  2-3 kb; 3’ | 25-50 kb  2.5-7 kb (exact size not assessed) | [9];[10] |
| β-thalassemia | 11p15.4 | *HBB* | 50 kb; 5’ | 30 kb deletion | [11] |
| Fascioscapulohumeral dystrophy (FSHD) | 4q35 | *FSHD* | 100 kb; 3’ | n D4Z4 deletions (n x 3.3 kb) | [12];[13]; [14] |
| Potocki-Shaffer syndrome | 11p11.2 | *ALX4* | >15 kb; 3’ | ~1.37 Mb deletion | [15] |
| Saethre-Chotzen syndrome | 7p21.1 | *TWIST1* | 5 kb; 3’ | translocation with 512 kb microdeletion | [16] |
| Leri-Weill dyschondrosteosis, idiopathic short stature | Xp22.33 | *SHOX* | 250-350 kb; 5’  30-530 kb; 3’  200 kb; 3’ | ring (X) with deletion of 700-900 kb of PAR1  12 microdeletions (<81 kb to 501 kb)  240-350 kb | [17]; [18]; [19] |
| Van Buchem disease | 17q21.31 | *SOST* | 35 kb; 3’ | 51.7 kb deletion | [20] |
| X-linked deafness with stapes fixation | Xq21.1 | *POU3F4* | 400 kb; 5’  900 kb; 5’ | microdeletions  overlapping microdeletions with 8 kb overlap | [21];[22] |
| X-linked recessive hypoparathyroidism | Xq26.3 | *SOX3* | 67 kb; 3’ | 23-25 kb deletion/340 kb insertion | [23] |

1. Lauderdale JD, Wilensky JS, Oliver ER, Walton DS, Glaser T (2000) 3' deletions cause aniridia by preventing PAX6 gene expression. Proc Natl Acad Sci U S A 97: 13755-13759.

2. Barbour VM, Tufarelli C, Sharpe JA, Smith ZE, Ayyub H, et al. (2000) alpha-thalassemia resulting from a negative chromosomal position effect. Blood 96: 800-807.

3. Romao L, Osorio-Almeida L, Higgs DR, Lavinha J, Liebhaber SA (1991) Alpha-thalassemia resulting from deletion of regulatory sequences far upstream of the alpha-globin structural genes. Blood 78: 1589-1595.

4. Higgs DR, Wood WG, Jarman AP, Sharpe J, Lida J, et al. (1990) A major positive regulatory region located far upstream of the human alpha-globin gene locus. Genes Dev 4: 1588-1601.

5. Beysen D, Raes J, Leroy BP, Lucassen A, Yates JR, et al. (2005) Deletions involving long-range conserved nongenic sequences upstream and downstream of FOXL2 as a novel disease-causing mechanism in blepharophimosis syndrome. Am J Hum Genet 77: 205-218.

6. Pop R, Conz C, Lindenberg KS, Blesson S, Schmalenberger B, et al. (2004) Screening of the 1 Mb SOX9 5' control region by array CGH identifies a large deletion in a case of campomelic dysplasia with XY sex reversal. J Med Genet 41: e47.

7. Benko S, Fantes JA, Amiel J, Kleinjan DJ, Thomas S, et al. (2009) Highly conserved non-coding elements on either side of SOX9 associated with Pierre Robin sequence. Nat Genet 41: 359-364.

8. Davies AF, Mirza G, Flinter F, Ragoussis J (1999) An interstitial deletion of 6p24-p25 proximal to the FKHL7 locus and including AP-2alpha that affects anterior eye chamber development. J Med Genet 36: 708-710.

9. McElreavy K, Vilain E, Abbas N, Costa JM, Souleyreau N, et al. (1992) XY sex reversal associated with a deletion 5' to the SRY "HMG box" in the testis-determining region. Proc Natl Acad Sci U S A 89: 11016-11020.

10. McElreavey K, Vilain E, Barbaux S, Fuqua JS, Fechner PY, et al. (1996) Loss of sequences 3' to the testis-determining gene, SRY, including the Y pseudoautosomal boundary associated with partial testicular determination. Proc Natl Acad Sci U S A 93: 8590-8594.

11. Driscoll MC, Dobkin CS, Alter BP (1989) Gamma delta beta-thalassemia due to a de novo mutation deleting the 5' beta-globin gene activation-region hypersensitive sites. Proc Natl Acad Sci U S A 86: 7470-7474.

12. Gabellini D, Green MR, Tupler R (2002) Inappropriate gene activation in FSHD: a repressor complex binds a chromosomal repeat deleted in dystrophic muscle. Cell 110: 339-348.

13. Jiang G, Yang F, van Overveld PG, Vedanarayanan V, van der Maarel S, et al. (2003) Testing the position-effect variegation hypothesis for facioscapulohumeral muscular dystrophy by analysis of histone modification and gene expression in subtelomeric 4q. Hum Mol Genet 12: 2909-2921.

14. Masny PS, Bengtsson U, Chung SA, Martin JH, van Engelen B, et al. (2004) Localization of 4q35.2 to the nuclear periphery: is FSHD a nuclear envelope disease? Hum Mol Genet 13: 1857-1871.

15. Wakui K, Gregato G, Ballif BC, Glotzbach CD, Bailey KA, et al. (2005) Construction of a natural panel of 11p11.2 deletions and further delineation of the critical region involved in Potocki-Shaffer syndrome. Eur J Hum Genet 13: 528-540.

16. Cai J, Goodman BK, Patel AS, Mulliken JB, Van Maldergem L, et al. (2003) Increased risk for developmental delay in Saethre-Chotzen syndrome is associated with TWIST deletions: an improved strategy for TWIST mutation screening. Hum Genet 114: 68-76.

17. Ellison JW, Tekin M, Sikes KS, Yankowitz J, Shapiro L, et al. (2002) Molecular characterization of a ring X chromosome in a male with short stature. Hum Genet 110: 322-326.

18. Benito-Sanz S, Thomas NS, Huber C, Gorbenko del Blanco D, Aza-Carmona M, et al. (2005) A novel class of Pseudoautosomal region 1 deletions downstream of SHOX is associated with Leri-Weill dyschondrosteosis. Am J Hum Genet 77: 533-544.

19. Fukami M, Okuyama T, Yamamori S, Nishimura G, Ogata T (2005) Microdeletion in the SHOX 3' region associated with skeletal phenotypes of Langer mesomelic dysplasia in a 45,X/46,X,r(X) infant and Leri-Weill dyschondrosteosis in her 46,XX mother: implication for the SHOX enhancer. Am J Med Genet A 137: 72-76.

20. Balemans W, Patel N, Ebeling M, Van Hul E, Wuyts W, et al. (2002) Identification of a 52 kb deletion downstream of the SOST gene in patients with van Buchem disease. J Med Genet 39: 91-97.

21. de Kok YJ, van der Maarel SM, Bitner-Glindzicz M, Huber I, Monaco AP, et al. (1995) Association between X-linked mixed deafness and mutations in the POU domain gene POU3F4. Science 267: 685-688.

22. de Kok YJ, Vossenaar ER, Cremers CW, Dahl N, Laporte J, et al. (1996) Identification of a hot spot for microdeletions in patients with X-linked deafness type 3 (DFN3) 900 kb proximal to the DFN3 gene POU3F4. Hum Mol Genet 5: 1229-1235.

23. Bowl MR, Nesbit MA, Harding B, Levy E, Jefferson A, et al. (2005) An interstitial deletion-insertion involving chromosomes 2p25.3 and Xq27.1, near SOX3, causes X-linked recessive hypoparathyroidism. J Clin Invest 115: 2822-2831.
